# Impact glasses from Belize represent tektites from the Pleistocene Pantasma impact crater in Nicaragua

**DOI:** 10.1038/s43247-021-00155-1

**Published:** 2021-05-17

**Authors:** Pierre Rochette, Pierre Beck, Martin Bizzarro, Régis Braucher, Jean Cornec, Vinciane Debaille, Bertrand Devouard, Jérôme Gattacceca, Fred Jourdan, Fabien Moustard, Frédéric Moynier, Sébastien Nomade, Bruno Reynard

**Affiliations:** 1 Aix-Marseille Université, CNRS, IRD, INRAE, UM 34 CEREGE, Aix-en-Provence, France; 2 IPAG Université Grenoble Alpes, CNRS, Institut de Planétologie et d’Astrophysique de Grenoble, Grenoble, France; 3 Centre for Star and Planet Formation, Globe Institute, University of Copenhagen, Copenhagen, Denmark; 4 Geologist, Denver, USA; 5 Laboratoire G-Time, Université Libre de Bruxelles, Brussels, Belgium; 6 School of Earth and Planetary Sciences, Curtin University, Perth, Australia; 7 Institut de Physique du Globe de Paris, Université Sorbonne Paris Cité, CNRS UMR 7154, Paris, France; 8 LSCE, CEA, UVSQ et Université Paris-Saclay UMR 8212, Gif–sur-Yvette, France; 9 Université de Lyon, ENSL, UCBL, CNRS, LGL-TPE, Lyon, France

## Abstract

Tektites are terrestrial impact-generated glasses that are ejected long distance (up to 11,000 km), share unique characteristics and have a poorly understood formation process. Only four tektite strewn-fields are known, and three of them are sourced from known impact craters. Here we show that the recently discovered Pantasma impact crater (14 km diameter) in Nicaragua is the source of an impact glass strewn-field documented in Belize 530 km away. Their cogenesis is documented by coincidental ages, at 804 ± 9 ka, as well as consistent elemental compositions and isotopic ratios. The Belize impact glass share many characteristics with known tektites but also present several peculiar features. We propose that these glasses represent a previously unrecognized tektite strewn-field. These discoveries shed new light on the tektite formation process, which may be more common than previously claimed, as most known Pleistocene >10 km diameter cratering events have generated tektites.

Four tektite strewn-fields have been recognized so far, all of them identified since the 1930’s:^1–4^ australasites, ivoirites, moldavites, and North American tektites (also called bediasites and georgiaites). Only the latter three strewn fields have been firmly connected to a known source crater (Bosumtwi, Ries, and the Chesapeake Bay, respectively), which all have diameters >10 km. The identification of the australasite source crater is still controversial despite recent studies, but its diameter is suggested to be >>10 km^5,6^. The specificities of the generation process of tektite versus other known impact glasses are not well understood, despite numerous sample studies and modeling. Tektite generation may be favored by high velocity (>20 km s^−1^) and low angle impacts in a soft water-rich surface, such as loess or soil^7–9^. Why only four out of the 78 known impact craters larger than 10 km in diameter^10^ are known to have generated tektites, including the putative crater responsible for the australasites, still remains an unsolved and challenging issue.

The criteria for differentiating between impact and volcanic glasses are their very low water content (compared to obsidians), the presence of high-temperature and/or high-pressure phases, and potential contamination by extra-terrestrial matter derived from the impactor. Tektites are characterized by the size of the strewn-fields (over 300 km wide except the Ivoirite case that is about 60 km wide) and the distance from the source crater (over 200 km and up to 5000 km or even 11,000 km including microtektites^11^). Other criteria include in particular their volatile depletion, the rarity of unmelted inclusions, low vesicularity, chemical homogeneity^3^, and a reduced character^12,13^ marked by the lack of Fe^3+^.

## Results

**Fig. 1:**
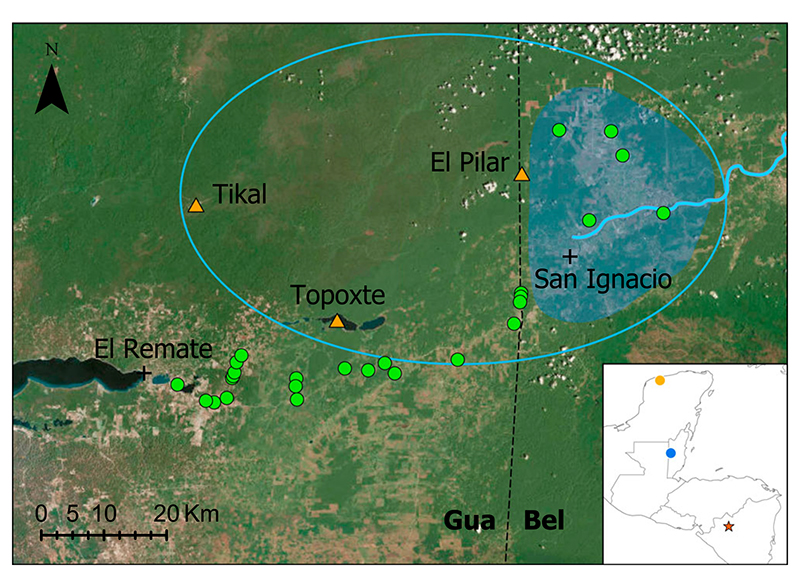
simplified map of the belizites strewn-field. simplified map of the belizites strewn-field (blue area) based on prospection in Belize, with Belize river highlighted by a thick blue line. Ellipse in thin blue line is the putative strewn-field including confirmed archeological findings. San Ignacio is at 17° 09’N and 84°04’W. Mayan sites with belizites findings (orange triangles) and sites visited by P.R. for this study (green circles). The inset map shows Central America with the location of the Pantasma crater (red star), belizite strewn-field (blue circle), and Dzibilchaltun Mayan site (orange circle).

In archeological excavations of the Mayan city of Tikal (North Guatemala; Fig. 1), tektite-looking glasses were identified^14,15^. Based on their andesitic composition (i.e., different from obsidian artifacts), low water content (≈80 ppm), and low Fe^3+^ concentration (from Mössbauer spectroscopy and magnetic properties) they were proposed to be tektites. J. Cornec (J.C.) engaged in geological mapping of the San Ignacio area of Central Belize, simultaneously discovered in natural outcrops numerous black glass specimens, which were confirmed to be similar in composition and age to the Tikal glass^16–18^, both dated around 800 ka. However, no detailed description of the Belize glass collection has been published yet. In this study, we describe this collection (hereafter named the J.C. collection) that represents more than 7000 specimens (total mass over 30 kg) and demonstrate that these glasses were ejected 530 km away from the 14 km diameter Pantasma impact crater recently discovered in Nicaragua^19^.

The geology of the area covered by Fig.1 is quite simple, with subhorizontal Eocene to Cretaceous marine limestones with minor interbedded shales^20^. To the SW (outside the map) outcrops the metamorphic and granitic basement of the Maya Mountains. The Belize strewn-field lays in a depression along the Belize River in the Cayo district. Superficial post-Eocene formations can be identified in the Cayo district, mainly the Red Bank formation, a gray-brown bentonitic clay (up to 20 m thick) interpreted as deriving from alteration of Oligocene-Miocene tephras, and post-Miocene gravels. Glass specimens are found on top of the Miocene clay below the soil layer (see supplementary Fig. 1), or within post-Miocene gravels. The large majority of finds derives from the northern part of the reported strewn-field.

**Fig. 2:**
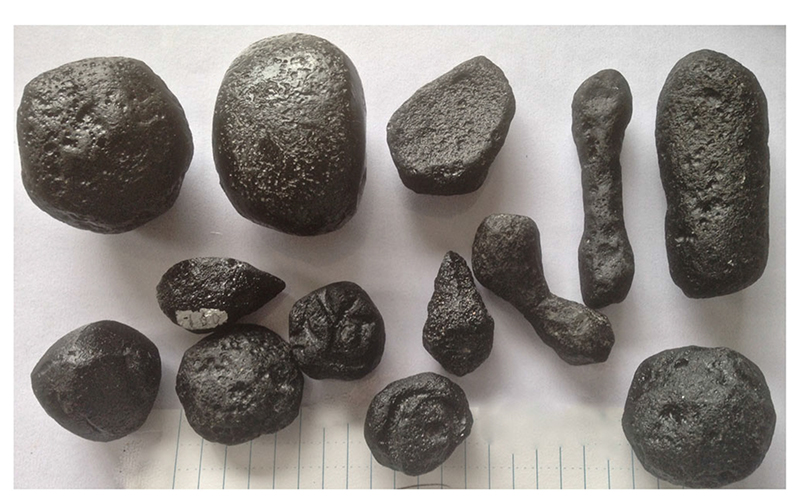
assortment of large belizites with typical shapes. assortment of large belizites with typical shapes. The longest sample is 7 cm long (one division is 5 mm).

The J.C. collection contains about 500 specimens with weights of >10 g, with a maximum single specimen mass of 103 g. The mass distribution of belizites may be interpreted as a fractal distribution, with an exponent −2.85, compared to −2.22 for a representative collection of ivoirites (see supplementary Fig. 2). This points toward a comparable high-energy fragmentation process^21^. Although the large majority of specimens are either spheroidal or irregular in shape, a number of well-defined splash forms were collected (Fig. 2, with elongated, teardrop, and dumbbell shapes). Some limited abrasion and pitting are observed. The specific gravity of standard bubble poor belizites is 2.51 ± 0.02 g cm^−3^ (average of 2 pools of samples), in the high range of known tektites^22^, linked to a lower SiO_2_ and higher Fe content than other tektites. By comparison, specific gravity measured in Pantasma glasses were 2.46 and 2.56^19^. Three more bubble-rich belizite samples yield specific gravity of 2.00, 2.13, and 2.34. Assuming a solid density of 2.51, this translates into a bubble content (closed porosity) of 20, 15, and 7%, respectively.

The porosity of most samples is low (<1% based on bubble counting) but a few samples are bubble-rich (one zone with up to 20% porosity). The magnetic susceptibility, measured on circa 4000 samples, is distributed in a very narrow range^23^ with a mean of 125 ± 4 × 10^−9^ m^3^ kg^−1^ excluding 31 more magnetic outliers (>200 × 10^−9^ m^3^ kg^−1^) that will be discussed later. Such a narrow distribution is typical of tektites^12,23^, as it indicates both the lack of Fe^3+^ and homogeneous iron content. Negligible Fe^3+^ is confirmed by synchrotron X-ray absorption near-edge structure spectra of 8 samples^24^. Major elements (Table 1) points toward andesitic composition on average, near the dacitic/trachytic limits: SiO_2_ and Na_2_O + K_2_O vary from 60.3 to 63.3 and 5.0 to 6.3 wt.%, respectively. Microprobe data on 6 individual samples were used to estimate variability on major elements, both between samples and within samples (Table 1). This variability is similar to what is observed for ivoirites, but lower than the one observed in other tektite strewn-fields^25,26^. Variability is significantly lower within than between samples. The good match between the scarce data for Tikal glass^14,15^ and our belizite data is shown in supplementary Fig. 3.

The water content measured by Fourier transform infrared spectroscopy on two specimens yields values of 53 and 114 ppm, in the low range for tektites^3^. It compares well to previous data from:^24^ 60–80 ppm. It is lower than the 240–280 ppm range determined in Pantasma glass^19^, in agreement with the fact that tektites are more depleted in volatiles than proximal glass.

**Fig. 3:**
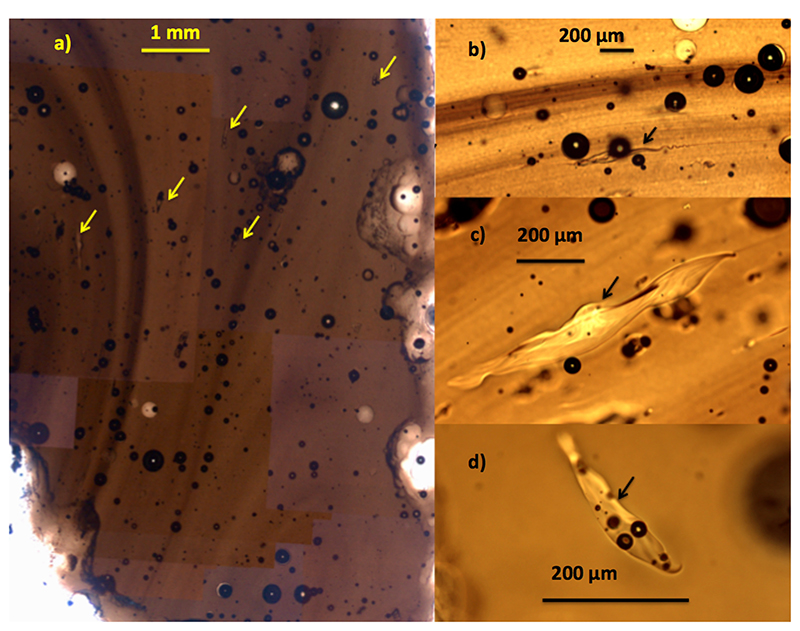
optical transmission images of lechatelierite. optical transmission images of lechatelierite (arrows), vesicles, and fluidality in belizites ≈1 mm thick sections; (**a**) full composite image; (**b**–**d**) selection of representative lechatelierites.

**Fig. 4:**
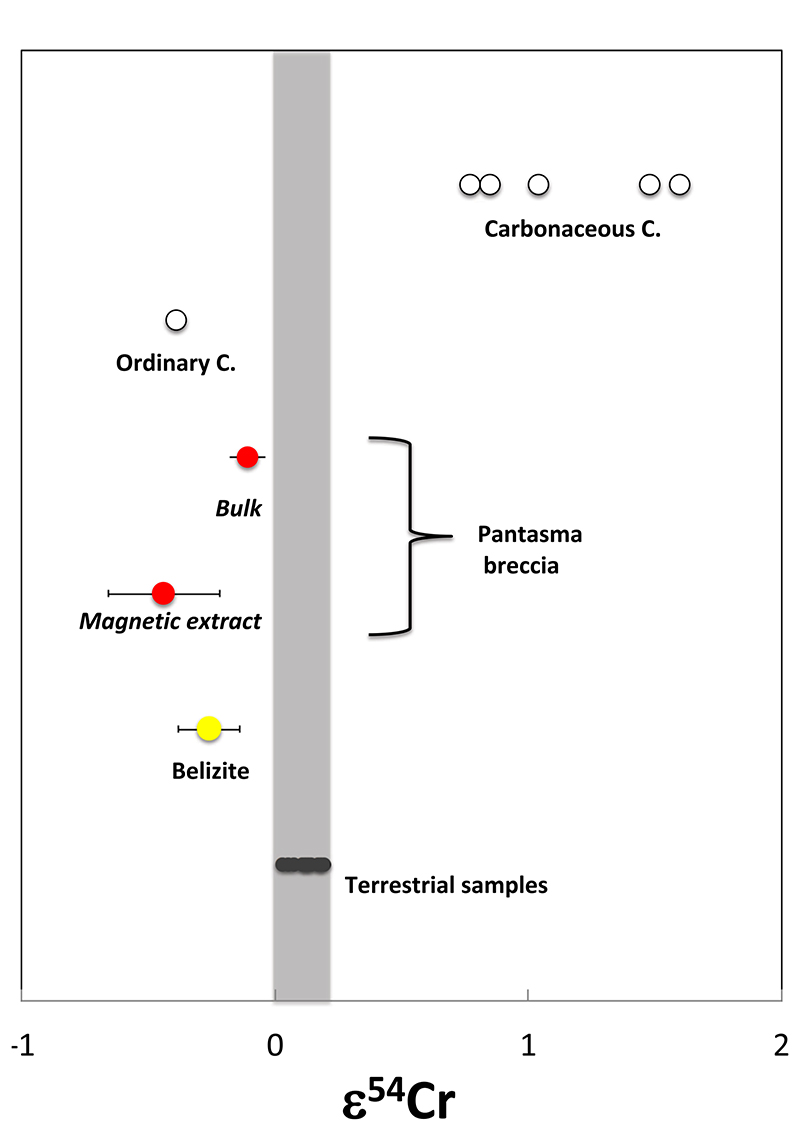
Cr isotopic data. ε^54^Cr data obtained on a belizite sample, the Pantasma breccia bulk (after^19^) and magnetic extract, compared to carbonaceous and ordinary chondrites^28^, as well as terrestrial rocks^29^. 2σ error bars shown.

Optical and back-scattered electronic microscopic investigations (Fig. 3) reveal fluidal textures typical of pure impact glasses, with no unmelted inclusions and limited content of spherical vesicles. The only common (still <1 vol.%) inclusions are pure SiO_2_ with smooth edges and often strong elongation, which is typical for the high-temperature silica glass lechatelierite^19^. Lechatelierite was further identified by Raman spectroscopy, along with a few α-cristobalite inclusions (Supplementary Fig. 4 and Note 1). The search for contamination by extra-terrestrial matter using chromium isotopic ratio analyses (e.g., ref. ^27^) yields clear evidence of contamination by an ordinary chondrite impactor: ε^54^Cr is −0.26 ± 0.12 and −0.39 ± 0.12 for a belizite sample and ordinary chondrites^28^, respectively (Fig. 4). Belizite data is clearly outside the terrestrial range: ε^54^Cr > 0.02^29^. Formation of belizites by impact melting is thus firmly grounded.

The current size of the glass strewn-field in Belize is small (<30 km, Fig. 1, Supplementary Note 2) and has not increased through time, despite intensive searches since 1990 by J.C. and other researchers, including locals trained by J.C. The inclusion in the inventory of impact glasses recovered in Maya excavations from Tikal^14,15^ may extend this size to 80 km. However, the discovery of such obsidian-looking glass in these archeological excavations may either be interpreted as being of local natural source or from the trade of this rare material from a Mayan city within the Belize strewn-field. The latter hypothesis is not presently supported by archeological studies as no evidence of reworking or presence in ceremonial deposits has been found. However, this eventuality cannot be discarded without further dedicated study. To test if belizites are present between Tikal and the Belize border, one of us (P.R.) performed a five days systematic search (Fig. 1) without making any new discoveries. Several tektite-lookalike gravels were collected near El Remate, but they were identified as cherts or volcanic rocks in the laboratory. However, the rate of discovery in the established Belize strewn-field is of the order of one sample per day. Belizite-like glass was reportedly^30^ found in Maya sites from El Pilar, Topoxte, and Dzibilchaltun (450 km further North in Yucatan, Fig. 1 insert). Therefore, it seems that the strewn-field defined in central Belize is relatively rich in belizites, whereas fewer belizites occur elsewhere, in Guatemala and possibly Yucatan.

### Pantasma crater as the source of belizites

**Fig. 5:**
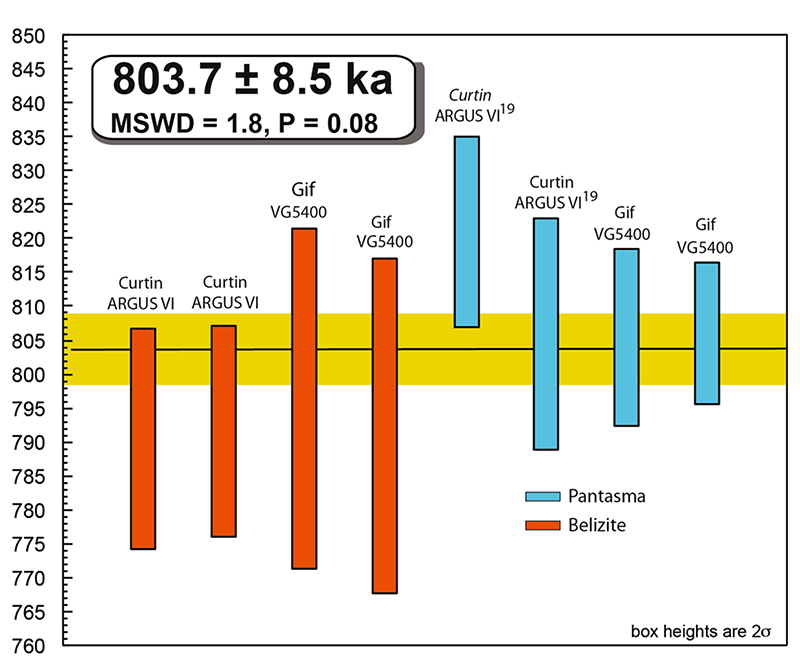
5 Ar/Ar ages. ^40^Ar/^39^Ar plateau ages with 2σ boxes obtained from the belizites and Pantasma impact glasses in two different laboratories (Curtin and LSCE Gif), together with the composite average in yellow. The first two Pantasma ages come from^19^.

Pantasma has been suggested as a candidate source crater for the belizites because of coeval ages with proximal impact glasses^19^. However, this hypothesis remains tied to published ages on belizites that are rather scattered and of low precision^16–18^. We, therefore, introduce four new high-precision plateau ^40^Ar/^39^Ar ages of belizite samples (Fig. 5), compared to the same number of ages for Pantasma glasses (implementing^19^ with two new ages). These ages are obtained in two laboratories using the same protocol and samples (see supplementary Fig. 5 and Supplementary Data 1 for spectra, isochrons, and full data). Average ages for belizite and Pantasma glass are 792.1 ± 9.2 ka (2σ; *n* = 4; Mean Squares Weighted Deviation (MSWD) = 0.05; Probability (*P*) = 0.98) and 809.1 ± 6.4 ka (*n* = 4; MSWD = 1.2; *P* = 0.30). We also tested if these age distributions could result from a single impact event by using a χ^2^ test on the entire dataset. Using the present eight ^40^Ar/^39^Ar plateau ages returns a P-value of 0.081 which is concordant, yielding a weighted mean age of 803.7 ± 8.5 ka (*n* = 8; MSWD = 1.8). The available ^40^Ar/^39^Ar data are thus fully compatible with belizites and Pantasma impact glasses being both produced from the same impact event.

**Fig. 6:**
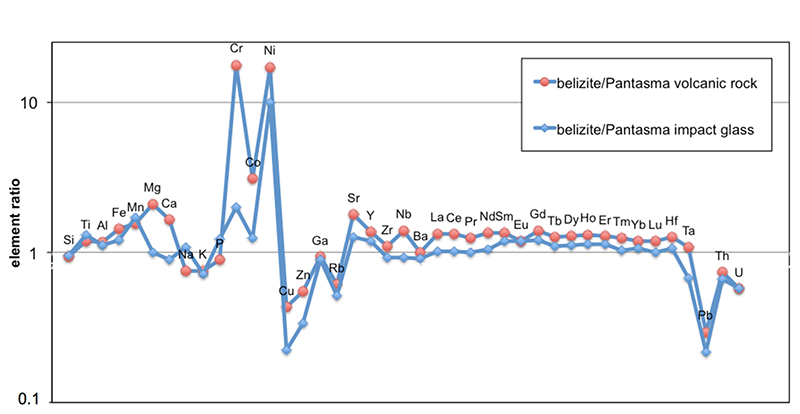
Normalized average composition of belizites. Average composition of belizites (see Supplementary Data 2) normalized to the average of the Pantasma volcanic rocks or impact glass^19^.

**Fig. 7:**
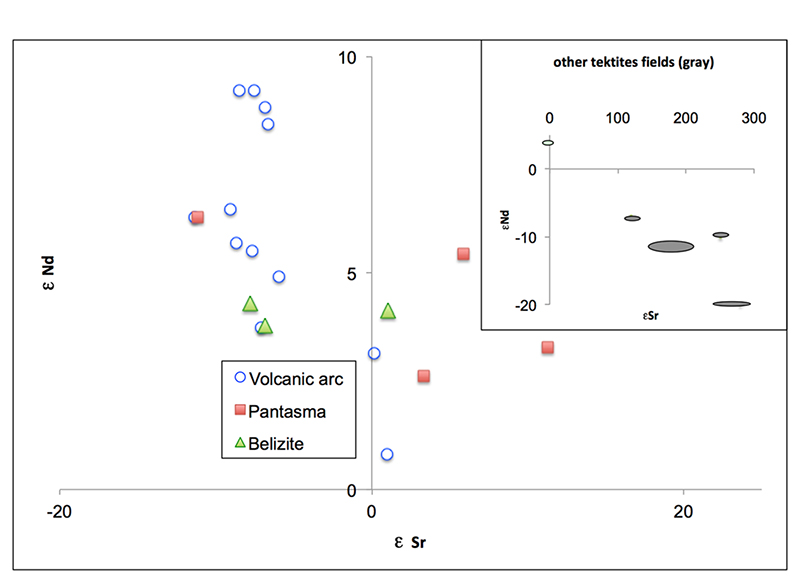
Nd versus Sr isotopic results. εNd versus εSr isotopic values for belizites compared to Pantasma rocks, soil, and glass, as well as Guatemala to Nicaragua volcanic arc data^32^. Insert plot: average data for the other tektite strewnfields in grey^11^.

Major and trace elements obtained on three 50 g pooled samples of belizites are highly homogeneous (supplementary data 2). Normalized to an average value for the Pantasma target rocks and impact glasses^19^, the belizites data show flat spectra close to 1 for most non-volatile and non-siderophile elements (Fig. 6), especially when compared to Pantasma glass. The clear enrichment in Cr, Ni, Co suggests extra-terrestrial contamination, which is confirmed by the analysis of Cr isotopic ratio (Fig. 4). A rough estimate for the amount of extraterrestrial contamination can be derived from a mixing model comparing average Pantasma rocks, belizite, and ordinary chondrite Ni and Cr contents^31^. Both elements provide the same value of contamination, 0.6%, which is likely an overestimate as Pantasma soils are also enriched in Ni and Cr of likely terrestrial origin^19^. Moderately volatile elements (Na, K, Cu, Zn, Rb, and Pb) are depleted, which is commonly observed in tektites^11^, resulting from the very high temperatures reached during their formation. Some differences occur in other elements (such as Ti, Mn, K, Sr, Th, U, for Pantasma glass), maybe due to the fact that the material that produced tektites may come from a different depth in the target than the analyzed Pantasma glasses and rocks. However, the overall match, particularly between belizites and proximal impact glasses from Pantasma, is consistent with their cogenesis. Isotopic ratios of radiogenic elements Sr and Nd are commonly used to trace the source of tektites^11,27^. Figure 7 (see Supplementary Table 1) shows that these ratios are similar in both the belizite and Pantasma glass and rocks. Moreover, the observed Sr and Nd ratios lay within the observed range for the volcanic arc in Nicaragua^32^, and are very different from the values for all other tektites^11^. Variable εSr values may tentatively be attributed to variable weathering effects, but we note that observed εSr variability is lower than for the previously known tektite strewn-fields^11^.

Pantasma impact glasses are rich in melted inclusions of Fe–Ti oxides with peculiar granular texture^19,23^. For the 31 belizite samples that are anomalously magnetic, one-third of them have χ > 2000 10 ^−9^ m^3^ kg ^−1^. For those samples, hysteresis shape and parameters are typical of magnetite. Saturation magnetization measured allows estimating pure magnetite equivalent content of 0.2 to 1%. A microscopic search in those samples revealed the presence of Fe–Ti oxide inclusions similar to those observed in Pantasma impact glasses (supplementary Fig. 6). This also supports a common origin, as well as the ordinary chondrite signature found in both Pantasma impact breccia PB5 and belizites (ref. ^19^ and Fig. 3). In fact, the magnetic extract from P5B breccia, with ε^54^Cr = −0.44 ± 0.22, appears more contaminated by extraterrestrial matter compared to the bulk P5B (ref. ^19^ and Fig. 4), thus reinforcing the evidence for extraterrestrial contamination in Pantasma breccia.

The numerous evidence for cogenesis between belizites and Pantasma impactites listed above thus supports the interpretation of coeval ^40^Ar/^39^Ar age determinations.

### Are belizites tektites?

The delineation of a specific type of impact glass called tektite was initially based on an empirical definition, i.e., a material alike the first three known tektite strewn-fields^1,2^. A more rational definition was proposed^3^ with criteria (Table 2 of ref. ^3^) being the occurrence in a sufficiently large strewn-field, away from source crater if known, the chemical homogeneity, water content <200 ppm, regular “spherically symmetric” shape, rare inclusions (mostly lechatelierite), low extra-terrestrial contamination, low heavy noble gases content and derivation from a continental surface. Our analysis of tektite literature raises further criteria: depletion in volatile elements, lack of Fe^3+^, regular splash forms with maximum mass >>10 g (allowing to discriminate tektites from “tektite-lookalike” impact glass, named tektoid^12^ such as irghizites and atacamaites, as well as Libyan desert glass). Apart from heavy noble gases content and derivation from a continental surface (discussed below), we have already shown that belizites fulfill all those criteria, with values well within the range of other tektite strewn-fields, and clearly different from other known non-tektite impact glasses.

For young enough impact glasses, derivation from the continental surface can be proved by significant atmospheric ^10^Be content^33–35^. Eight belizite samples yield an average ^10^Be content (corrected to the time of impact) of 9.1 ± 2.1 Mat g^−1^ (Supplementary Table 2), not significantly different from the mean from 7 samples at 12 ± 5 Mat g^−1^ reported in^36^. It is significantly lower compared to the ivoirites and australasites: 35 ± 7 and 143 ± 50 Mat g^−1^, respectively.

Alternative sources for the ^10^Be content of belizite may be in situ production, and oceanic sediment recycling in subduction-related volcanics. However, these sources are estimated to correspond to less than 1 Mat g^−1^ (see discussion in supplementary note 3). The average belizite value lies between the values measured from two Pantasma soil samples (290 ± 8 Mat g^−1^) and two Pantasma impact glasses (0.6 ± 0.1 Mat g^−1^). This indicates that although the belizites are derived from an upper level of the target rocks compared to the Pantasma impact glasses, they contain a smaller fraction of surface soil (<few %) than the ivoirites and australasites, which is possibly due to the rugged terrain on which the impact occurred^19^. Concerning heavy noble gases, our Ar/Ar experimental protocol does not allow us to compute reliable ^36^Ar content. However, this criterion seems somehow redundant with water and other volatile elements contents and not able to distinguish Muong Nong tektites from non-tektite impact glasses^37^. Belizites thus share most characteristics of tektites. They closely match ivoirites in many aspects: size and shape of specimens, strewn-field size, distance to the crater, crater diameter, presence of minor ordinary chondrite contamination.

## Discussion

The proposed belizites-Pantasma crater couple is the fourth known tektite-crater couple, and presently the youngest. The suggested synchronism of this event with the australasites^18^ is not supported by our composite age for belizite-Pantasma, at 803.7 ± 8.5 ka, compared to the most recent high-precision age for australasite, at 788.1 ± 2.5 ka^38^; both ages were obtained in the same laboratories and are reported with 2σ error bars. Besides the observed common features among the four tektite-crater couples, plus the australasite strewn-field, the Central American couple displays a number of peculiarities. It is the only strewn-field to show a mantle signature due to its derivation from volcanic arc target rocks (Fig. 7 and ref. ^39^). The target near-surface origin of the ivoirites and australasites, demonstrated by high ^10^Be content^32–34^, is less prominent in the belizites. A final peculiarity is the presence of cristobalite, previously described only in layered tektites from Indochina^40^. These peculiarities, together with the lack of noble gases data, suggest that our interpretation of belizites as tektites requires further confirmation to be wholly accepted.

A common idea about tektites is that they may have formed by unusual process^7–9^, based in particular on the low number of known strewn-fields compared to known craters >10 km: presently 4 out of 78 (or 14 if one restricts the list to well-dated post K/T craters: Table 2). One may cite more distal microtektite like ejecta (from Popigai, Chicxulub, and several Precambrian spherule layers^4^) but the list of tektite strewn-fields has been restrained to younger than K/T and presence of macroscopic material^8^. Considering only young craters is partly motivated by strewn-field preservation issues. Indeed, erosion will rapidly destroy the continental surface hosting the strewn-fields, unless it is rapidly buried under post-impact sediments, as in the case of moldavites and bediasites-georgiaites. If we restrict the comparison to the known four Pleistocene well-dated large cratering events^10,38,41^, i.e., australasites, Pantasma, Zhamanshin, Bosumtwi, this figure becomes 3 out of 4. For the rationale to assume that the australasite event implies an impact crater >10 km diameter, one may refer to refs. ^5,6,42^. The only exception to the rule would be Zhamanshin but its actual diameter is quite debatable: the reported gravity anomaly^43^ is only 6 km in diameter, similar to the diameter of the crater-like depression with steep rims visible on the topography (supplementary Fig. 7). The published 14 km diameter is a poorly defined circular feature that may correspond to a pre-impact topography. Pleistocene events point toward tektite production being the rule in large-enough craters rather than the exception, based on the low probability of finding tektites from craters that are much older than Pleistocene, due to preservation issues. If tektite production was on average characterizing less than 10% of large impacts, the probability to obtain a sequence of 3 out of 4 events is less than 3 per mil. The next-youngest well-dated large craters include El’gygytgyn, at 3.6 Ma, and the tektite producing Ries crater at 14.81 Ma^10,44^. Therefore, there is still a majority (4 out of 6) of large tektiteproducing craters since the Middle Miocene. The putative tektite strewn-fields from Zhamanshin and El’gygytgyn craters would be located in remote semi-desertic areas, and hence may have remained undiscovered. An independent clue for the rather “normal” production of the tektite is the observation that the most common type of impactor, ordinary chondrite, is responsible in the three cases for which it has been identified: belizites, ivoirites^45^, and possibly australasites^46^. This agrees with the theoretical and experimental arguments that the type of projectile does not influence melt production^7,8^.

The evidence from the proposed central American tektitecrater couple can be compared to the previously known couples or strewn-fields, and allows making two predictions: (1) the analogies between the belizites and ivoirites strongly suggest that thousands of ivoirites may remain to be found in this very poorly explored strewn-field^2,41^ where only a few hundred tektites are currently reported; (2) microtektites ejected from the Pantasma crater may be found within a few thousands of km distance from the crater, i.e., within oceanic sedimentary cores, as is the case for the ivoirites strewn-field^47^. This may help to confirm our findings that the australasites are slightly younger than belizites, based on the climatostratigraphic positions of the respective microtektite layers in the sedimentary cores. We emphasize that searching for microtektite layers, or other distal impact material (e. g., clinopyroxene and impact glass spherules^4^) connected to other well-dated large craters may help to overcome the preservation issue for macroscopic tektites and confirm our proposition for a ubiquitous tektite production process for large impacts. As an example, a microtektite layer dated 56 Ma has been proposed recently^48^.

## Methods

Thin sections of representative rocks were prepared for optical microscopy, while polished thick sections of the glass were examined in CEREGE using a Hitachi S3000-N scanning electronic microscope (SEM), operated at 15 kV, and fitted with a Bruker energy dispersive spectrometry (EDS) microanalysis system. Additional higher-resolution images were obtained at Center Pluridisciplinaire de Microscopie électronique et de Microanalyse (CP2M), Marseille, with a Zeiss Gemini 500 field-emission gun SEM operated at 15 kV and fitted with an EDAX EDS microanalysis.

Further microanalyses were obtained by electron microprobe analysis using a CAMECA SX-100 at the Center de Microanalyse de Paris VI (CAMPARIS).

Double-polished sections were prepared from two belizite samples in order to determine water content through transmission measurements. Samples were polished using SiC disks under ethanol until they were optically thin through the spectral range of interest and their thickness was measured using a Mitutoyo Digimatic micrometer. Water content was determined using Fourier transform infrared (FTIR) transmission microscopy with a VERTEX V70 spectrometer coupled to a Hyperion 3000 infrared microscope (IPAG, Grenoble; see Supplementary Method 1).

Raman spectra of SiO_2_ rich inclusions in several belizite polished sections were obtained using a LabRAM HR800 Evolution spectrometer that has a confocal Czerny-Turner geometry and a laser source of 532 nm in wavelength. Each spectrum was acquired with a power of 10 mW, and 25 accumulations of 5 to 15 s. Gratings with 600 groove/mm were used in order to cover the frequency range 60 to 1300 cm^−1^.

Magnetic susceptibility (χ) measurements of glass samples of J.C. collection were obtained in Denver using the portable SM150 susceptibility meter (working in a field of 1 kHz frequency and 320 A m ^−1^ intensity) that has a sensitivity better than 10^−9^ m^3^ kg^−1^ for sample mass >5 g. This mass threshold was obtained in the case of small samples by pooling a sufficient number of them in the holder, thus explaining that the histogram presented in^23^ is built from 1000 measurements made on >4000 samples. Hysteresis measurements were performed on the high susceptibility samples using a Micromag VSM in CEREGE. Specific gravity was determined using a precision scale and volume determination by helium pycnometry with a Quantachrome instrument in CEREGE.

Major and trace element analyses were performed on Pantasma rocks and glasses at the Laboratoire G-Time at the Université Libre de Bruxelles. For each sample, approximately 50 mg of crushed material was dissolved by alkaline fusion using an ultrapure (>99.999%) mixture of 4:1 lithium metaborate and tetraborate. Major elements were measured using a Thermoscientific ICaP inductively coupled plasma atomic emission spectroscopy (ICP-AES) instrument (see Supplementary Method 2). For trace elements, the concentrations were measured on an Agilent 7700 quadrupole ICP-mass spectrometer. For belizites, analysis was performed by ALS Minerals inc. (Reno, USA) using for minor elements ICPMS after lithium borate fusion and for major elements XRF after fusion. Three batches were analyzed, each circa 30 g and made of a pool of cleaned tektites.

For isotope measurements, around 100 mg of samples were digested by digesting subboiled concentrated 1:3 mixture of HF and HNO_3_ and subboiled concentrated HCl after evaporation. After complete digestion, an aliquot was separated for Sm-Nd spike, another one for Rb/Sr measurements, and the last one for Sr isotopes. Strontium was purified on Sr-Spec resin using HNO_3_. The rare earth elements from the remaining solutions were purified using first cationic resin AG50X8 in HCl, and then Nd was purified from Ce and Sm using HDEHP resin, also using HCl. The spiked cuts followed the same chemical purification. The Nd and Sr purified cuts were both measured on the Nu-Plasma 2 multi-collection ICP-MS at Laboratoire G-Time (see Supplementary Method 3).

For Cr isotope analysis, 30 mg of belizite sample BLZ3, as well as of the magnetic extract from P5B breccia (bulk fine fraction measured in ref. ^19^) was dissolved in a 3:1 mixing of HF-HNO_3_ in Teflon bombs at 140 °C. ^53^Cr/^52^Cr and ^54^Cr/^52^Cr isotope ratios were measured by Thermal-Ionization Mass-Spectrometry (TIMS) Fisher Scientific Triton at the Center for Star and Planet Formation, University of Copenhagen (see Supplementary Method 4).

For Ar/Ar geochronology in both laboratories, glass fragments were handpicked from the 215–315 μm fraction of glass under the binocular stereomicroscope and were thoroughly rinsed in distilled water and loaded into an aluminum disc for irradiation. In Curtin Argon laboratory Ar isotopes were measured in static mode using a low volume (600 cc) ARGUS VI mass spectrometer from Thermofisher© set with a permanent resolution of ~200. In LSCE argon isotope measurements were undertaken at the GIF ^40^Ar/^39^Ar facility (CEA Saclay University Paris Saclay and Versailles St Quentin, France). Argon isotopes were successively measured using a VG 5400 mass spectrometer equipped with one electron multiplier (Balzer SEV 217 SEN) associated with an amplifier and discriminator module (Micromass MA 3971/1). (see Supplementary Method 5)

^10^Be content has been analyzed in 10 samples (6 belizites, 2 Pantasma soil, and 2 Pantasma glass) by accelerator mass spectrometry (AMS) at the French AMS national facility ASTER, housed at CEREGE, Aix en Provence, France (see Supplementary Method 6).

## Supplementary Material

The online version contains supplementary material available at https://doi.org/10.1038/s43247-021-00155-1.

Data 1

Data 2

Description of supplementary files

Methods

## Figures and Tables

**Table 1:** major elements data.

Elts	Na_2_O	MgO	SiO_2_	Al_2_O_3_	K_2_O	CaO	FeO
Mean wt.	3.6	1.8	62.0	17.2	2.0	4.7	6.4
% MPA							
Min wt.%	2.7	1.5	60.3	16.1	1.7	4.0	5.5
Max wt.%	4.2	2.1	63.3	17.9	2.4	5.4	7.4
Mean wt.	3.76	1.84	61.73	16.95	1.92	3.79	7.01
% XRF							
% variability							
Belizites^a^	37.3	25.4	4.6	10.1	28.8	26.6	25.3
Belizites^b^	15.6	13.0	1.2	3.4	6.8	5.8	9.6
Belizites^c^	23.3	18.1	2.3	6.1	9.1	9.7	20.6
Ivoirites	36.9	32.8	3.3	7.6	17.9	55.9	11.3
Australasites	60.3	83.5	18.7	49.7	52.3	81.1	58.6
Moldavites	-	58.8	12.0	47.0	41.5	76.0	69.1
Bediasites-georgiaites	45.7	-	14.0	46.0	36.3	-	68.2

Average major elements (>1 wt.%, i.e., excluding Mn and Ti) analyzed by microprobe (MPA) on 6 belizite samples (3 to 24 individual measurements were first used to produce sample average) with the indication of minimum and maximum values. The next line indicates average values obtained by X-ray fluorescence (XRF; see supplementary data 2). Between samples relative variability in %, (1-min/max) ×100, is indicated for belizites, as well as average or maximum intra sample variability (^a, b, c^ respectively). Between samples variability in other tektite strewn-fields after^25,26^ (again only for elements >1 wt.% on average)

**Table 2:** List of well-dated post K/T cratering events >10 km diameter.

Crater	Diameter (km)	Age (Ma)	Melt type		
			1	2	3
Pantasma	14	0.8	x	x	
Australasian	? (>10)	0.8	x		
Zhamanshin	14?	0.9		x	
Bosumtwi	10.5	1.1	x	x	
El’gygytgyn	18	3.6		x	x
Ries	24	15	x	x	x
Haughton	23	23			x
Logoisk	15	30			x
Chesapeake	40	35	x		x
Pogigai	90	36	*	x	x
Mistastin	28	38			x
Montagnais	50	51			x
Kamensk	25	51			x
Marquez	12	58			

Well-dated post K/T cratering events >10 km diameter (postulated for Australasian strewn-field) ordered by age^10^. Melt type encountered indicated by a cross (x): (1) tektite, (2) proximal glass in the ejecta, (3) in-situ glass, and melt in the crater floor. For glass in suevite, the distinction between 2 and 3 may be problematic.

* The presence of long-distance ejecta as clinopyroxene spherules^4^. ? means unknown in the first cell (?(>10)) and questionable in the second cell (14?)

## Data Availability

Ar/Ar raw data is available in Supplementary Data 1, and geochemical data in Supplementary Data 2. Sr, Nd, and Be isotopic data are available in Supplementary Tables 1 and 2. All data are also available at DOI: 10.5281/zenodo.4633592.
