# Supplementary material for: Impact glasses from Belize represent tektites from the Pleistocene Pantasma impact crater in Nicaragua
Source: Commun Earth Environ. Author manuscript; Available in PMC 2021 Aug 17. (PMC7611520; doi:10.1038/s43247-021-00155-1)

## supplementary methods

## Method 1: Water content

The infrared light is focused and collected through two Cassegrain objectives and the area through which the water content is measured is 100 μm by 100 μm. Infrared measurement were obtained in the 4000-1000 cm-1 spectral range and water contents were estimated by the intensity of the –OH fundamental stretching band at 3550 cm-1 (using a molar absorptivity coefficient of 67 L/mol[1]). Due to uncertainty in the measurement of sample thickness (10 μm) a relative uncertainty of 5 % is estimated given the typical final size of the analyzed sample (100s of μm). Note that this approach was validated through measurement of synthetic basaltic glasses[2].

## Method 2: Elemental geochemistry

Samples were doped with Y as internal standard and the calibration curve was obtained on synthetic standards. International standards BCR-2, BHVO-2, AGV-2 and BIR-1 were used for monitoring accuracy. The relative difference between the accepted and measured values was lower than 5% for individual elements and lower than 2 % for the total wt. %. Loss on ignition (LOI) was measured after 1 hour at 1000°C and corrected for Fe content. For trace elements, the concentrations were measured on an Agilent 7700 quadrupole ICP-mass spectrometer. Samples were doped with In as internal standard and the calibration curve was obtained on synthetic standards. External reproducibility based on natural duplicate analyses is generally lower than 10% (2 relative standard deviation RSD), except for Cr, Ni and Cu (< 20% 2 RSD). International standards BCR-2, AGV-2 and BHVO-2 were used to monitor accuracy and yielded results within the accepted range.

Average data with s.d. is reported in supplementary table 2 for three pooled belizites samples, five Pantasma rocks (P4, P6, P9, P11 and P15) and two glasses (P1 and P2; full data already published in [3]).

### Method 3: Sr and Nd isotopes

Neodymium isotopes were measured in dry mode using an Aridus 2 dessolvator with solution at 25 ppb. The samples were bracketed with the Rennes Nd standard and corrected for the accepted value of the standard (0.511963 [4]). The external reproducibility based on 30 standard measurements is better than 29 ppm. The Nd and Sm spiked aliquots were measured afterwards and spike deconvolution used the same formulation as in [5]. For Sr isotopes measurements, the samples were measured in wet mode, at 400 ppb. Samples were bracketed with the NBS987 Sr standard and samples were corrected according to the accepted value (0.710248 [6]). The external reproducibility based on 18 standard measurements is better than 27 ppm. Finally, Rb/Sr ratios were measured on the Agilent 7700 ICP-MS by using a calibration curve made of gravimetrically prepared Rb/Sr solutions.

Two samples from belizite were measured, BLZ3 and BLZ6, and from Pantasma one glass (P1), two rocks (P6 and P15) and one soil (P17). Details on these Pantasma samples can be found in [3]. In the supplementary table 1 and in Fig.6 is also indicated for comparison the data published in abstract form by [7]. This work has also reported Os isotopic ratio, compatible with mantle derived origin.

### Method 4: Cr isotopes

The chemical separation was adapted from [8] and described in detail in [9]. It includes three separation steps of Cr on cationic exchange resin AG50W-X8 and was conducted at the Institut de Physique du Globe de Paris. Measurement used a filament

exhaustion sample standard bracketing approach following the method described in [10]. Chromium isotopic data are reported using the ε-unit, which represents the relative deviation in parts per 10,000 of $^{53}Cr/^{52}Cr$ and $^{54}Cr/^{52}Cr$ ratios from a terrestrial standard (NIST SRM 3112a Cr standard; see supl. Table B). In addition to breccia sample P5B, a USGS geostandard, BHVO-2, was analyzed during the same session to check for the accuracy of the method.

**Method 5: Ar/Ar geochronology**

For Curtin laboratory measurements the glass samples were irradiated for 3 hours alongside FCs standards[11], for which an age of 28.294 Ma (± 0.13%) was used[12]. The discs were Cd-shielded (to minimize undesirable nuclear interference reactions) and irradiated in the Oregon State University nuclear reactor (USA) in central position. The $^{40}Ar/^{39}Ar$ analyses were performed at the Western Australian Argon Isotope Facility at Curtin University. Three aliquots of glass with weight ranging from about 10 to 40 mg were step-heated using a continuous 100 W PhotonMachine© $CO_2$ (IR, 10.4 μm) laser fired on the glass during 60 seconds per step. Each of the standard crystals was fused in a single step. The gas was purified in an extra low-volume stainless steel extraction line of 240cc and using one SAES AP10 and one GP50 getter.

In LSCE Argon laboratory one belizite sample (JC collection) and one Pantasma impact glass sample (PV1) were irradiated for 2 hours in the Cadmium-Lined In-Core Irradiation Tube (CLICIT) Oregon State University TRIGA reactor (USA). Interference corrections were based on the nucleogenic production ratios quoted in [12]. After irradiation, two ~50 mg aliquots of each sample were loaded into a copper sample holder and placed within a laser sample chamber fitted with Cleartan© viewport.

Detailed analytical procedures can be found in [13]. Each aliquot was incrementally heated using a 25 Watts Synrad $CO_2$ laser. Extracted gases for each step were purified for 10

minutes using two hot AP 10 and two GP 50 getters (ZrAl). Each argon isotope measurement consisted of 20 cycles of peak-hopping. The neutron fluence (J) value for each sample was calculated using co-irradiated Alder Creek sanidine standard (ACs : 1.1891 Ma, equivalent to an FCs standard age of 28.294 Ma [59]) and a $^{40}$K total decay constant of [14]. J-values are the followings: Belizite = 0.00052333 ± 0.00000418 (2σ), Pantasma = 0.00052417 ± 0.00000420 (2σ). The mass discrimination was monitored by analyses of air pipette aliquots throughout the sample analyses, relative to a $^{40}$Ar/$^{36}$Ar atmospheric ratio of 298.56 ± 0.32[15]. 10 minutes blank measurements were performed after every 2-3 unknown analyses. All blanks are presented in the extended data table 1.

The complete LSCE dataset can be found in Extended data Table 1. $^{40}$Ar/$^{39}$Ar step heating results are presented in Extended data Fig. 6a as age spectra and inverse isochron plots. The plateau ages of each step-heated aliquot are presented, as well as a weighted mean age combining the two plateau ages (Extended data Fig. 6a). Combined inverse isochron ages from the 2 aliquots of the belizites and Pantasma (PV1) impact glass samples are presented (Extended data Fig. 6a). All the step-heated age spectra (n=4) are yield robust plateau ages with 100% of the total $^{39}$Ar, acceptable MSWD (0.02 to 0.23) and P values (0.98 and 1.0). The inverse isochron ages are within 2σ errors of the plateau ages, with acceptable Sf values, and initial $^{40}$Ar/$^{36}$Ar ratios that are within 2σ uncertainties of the atmospheric $^{40}$Ar/$^{36}$Ar ratio, suggesting no excess $^{40}$Ar component is present in these samples. (Supplementary Data Fig. 6 and supplementary Table 2).

**Method 6: $^{10}$Be preparation and measurement**

A carefully weighed mass spiked with of a 3025 ppm ± 9 ppm in-house $^{9}$Be carrier[16] was totally dissolved in 48% hydrofluoric acid and fumed in a PTFE beaker until dry. The precipitate was dissolved with nitric acid and the Be was purified by solvent extractions and alkaline precipitations. After being oxidized at 800 °C for 1 hour,

the BeO was mixed with niobium powder. All measurements were standardized against the in-house STD11 standard[17]. Analytical uncertainties (reported as 1σ) include uncertainties associated with AMS counting statistics, chemical blank measurements, and AMS internal error (0.5%).

## supplementary notes

### Note 1: Raman spectroscopy

Lechatelierite and cristobalite were identified, and were often present at the same time in a given sample, if not in a single silica inclusion within the glass matrix with complex composition (Supplementary Fig. 4). Lechatelierite shows the broad peaks of amorphous structure and the narrower D1 and D2 features at 498 and 604 $cm^{-1}$ that are typical of pure $SiO_2$ glass, whose relative intensities are similar to thermally processed silica glass[18]. Cristobalite shows similar peaks to those observed for synthetic polycrystalline samples of α-cristobalite, the form stable at ambient temperature, with prominent features at 110, 228, and 415 $cm^{-1}$, and smaller peaks at 272, 284, 780, 791, 1075, and 1193 $cm^{-1}$ [19]. α-cristobalite is the quench product of β-cristobalite, the high-temperature low-pressure silica polymorph stable above 1470°C and below 1713°C where it melts. In the studied belizite samples, melted silica either crystallized to cristobalite or quenched to glass (lechatelierite) depending on the cooling rates of the tektites.

### note 2: Field work and sampling

All samples of belizites analyzed come from the collection of J.C., obtained through thirty years of field work in Belize, either by personal prospection by foot on favorable terrains, or by training local cultivators and buying their finds periodically.

The contour of the circa 30 km large Belize strewn-field in Fig.1 is the results of thousands of hours of prospection. On favorable area one to a few tektite per day can be found. PR, after a training on the main strewnfield with JC (visiting the green points in Fig.1), and visiting Tikal and Topoxte Maya sites, spend 5 full days searching for tektite in Guatemala (search points in Fig.1) without finding a single tektite (only tektite-looking gravels). Since, a person from Guatemala working with JC brought him a tektite-looking pebble found in El Remate, i.e. 25 km south of Tikal, that was later identified as a weathered volcanic rock. Including the other Maya sites where G. Braswell reported findings similar to Tikal glass, a conservative approach for the minimum size of the strewn-field is to include the whole area from Tikal to the Belize prospected area, and exclude the north Yucatan Dzibiltchaltun find. Indeed, we have no documentation on this material (chemical analysis, visual aspect), and we prefer to consider the identification of this material as a belizite not yet fully proven. In particular it could be related to the nearby Chixculub crater. Therefore, the minimum size of the strewn-field would be about 80 km. This is nearly identical to the size reported for the ivoirite strewn-field (e.g. [2,40]).

All rock, soil and glass samples analyzed from Pantasma come from sampling made in 2016 and reported in [3]. In 2016, only 3 impact glasses pebbles were found by chance in the river gravels near the crater center. In 2020 a new prospection along the river allowed to find five new impact glass samples, similar to the 2016 ones. One of them has been investigated in[20].

**Note 3:** $^{10}$Be **results**

Instead of a small target soil contamination, one may propose two alternative origins for the $^{10}$Be measured in belizites:

1) in situ production since fall in Belize. In situ production over 0.8 Ma is at most 1.8 Mat/g, assuming the sample remained exposed at the surface for this whole duration. Field observations suggest on the contrary that samples remained under a soil or surface formation cover of the order of one meter on average, corresponding to about one third of the maximum production.
2) Presence of $^{10}Be$ in the volcanic rock due to subduction processes. Indeed contamination by oceanic sediments produce magma rich in $^{10}Be$. [21] have measured from 1 to 25 Mat/g (average 10 ± 7) in present day lava from Nicaragua. However, as Pantasma lava[19] are likely older than 10 Ma ( and not younger than 5 Ma), the remaining $^{10}Be$ at 0.8 Ma is less than 1.2 and likely about 0.1 on average. This estimation is conforted by the near zero content measured in Pantasma glass.

**Supplementary reference list**


1 Stolper E. Water in silicate glasses: An infrared spectroscopic study. *Contrib. Mineral. Petrol.* **81**, 1–17 (1982).

2 Rapin W., et al. Quantification of water content by laser induced breakdown spectroscopy on Mars. *Spectrochimica Acta Part B Atomic Spectrosc*opy **130**, 82-100 (2017)

3 Rochette, P. et al. Pantasma: a Pleistocene circa 14 km diameter impact crater in Nicaragua. *Meteorit. Planet. Sci.* **54**, 880-901 (2019).

4 Chauvel, C., & Blichert-Toft, J. A hafnium isotope and trace element perspective on melting of the depleted mantle. *Earth Planet. Sci. Lett.* **190**, 137–151 (2001).

5 Debaille, V., Brandon, A. D., Yin, Q.-Z., & Jacobsen, B. Coupled 142Nd-143Nd evidence for a protracted magma ocean in Mars. *Nature* **450**, 525–528 (2007).

6 Weis, D. et al. High-precision isotopic characterization of USGS reference materials by TIMS and MC-ICP-MS. *Geochemistry, Geophysics, Geosystems* **7(8)** https://doi.org/10.1029/2006GC001283 (2006).

7 Koeberl, C. & Schulz, T. Os isotopic analysis of tektite-like glasses from Belize show a volcanic provenance but no extraterrestrial component. 47th Lunar and Planetary Science Conference, LPI Contribution No. 1903, p.1654 (2016).

8 Trinquier A., Birck J. -L., and Allègre C. J. Widespread 54Cr heterogeneity in the inner solar system. *The Astrophysical Journal* **655**, 1179 (2007).

9 Mougel B., Moynier F., Göpel C. Chromium isotopic homogeneity between the Moon, the Earth, and the enstatite chondrites. *Earth Planet. Sci. Lett.* **481**, 1-8 (2018).

10 Van Kooten, E., et al. Isotopic evidence for primordial molecular cloud material in metal-rich carbonaceous chondrites. *Proc. Nat. Acad. Sci.* **113**, 2011-2016 (2016)
11 Jourdan F. and Renne P. R. Age calibration of the Fish Canyon sanidine 40Ar/39Ar dating standard using primary K-Ar standards. *Geochim. Cosmochim. Acta* **71**, 387-402 (2007)
12 Renne P. R., Balco G., Ludwig K. R., Mundil R. and Min K. Response to the comment by W.H. Schwarz et al. on "Joint determination of K-40 decay constants and Ar-40*/K-40 for the Fish Canyon sanidine standard, and improved accuracy for Ar-40/Ar- geochronology" by PR Renne et al. (2010). *Geochim. Cosmochim. Acta* **75**, 5097-5100 (2011).
13 Nomade S., Gauthier A., Guillou H., Pastre J-F. 40Ar/39Ar temporal framework for the Alleret maar lacustrine sequence (French Massif-Central): Volcanological and paleoclimatic implications. *Quaternary Geochronology* **5**, 20-27 (2010).
14 Niespolo, E.M., Rutte, D., Deino, A., Renne, P.R. Intercalibration and age of the Alder Creek sanidine $^{40}Ar/^{39}Ar$ standard. *Quat. Geochronol.* **39**, 205-213 (2017).
15 Lee, J.Y., Marti, K., Severinghaus, J.P., Kawamura, K., Hee-Soo, Y., Lee, J.B., Kim, J.S. A redetermination of the isotopic abundances of atmospheric Ar. *Geochim. Cosmochim. Acta* **70**, 4507-4512 (2006).
16 Merchel, S., et al. Towards more precise $^{10}Be$ and $^{36}Cl$ data from measurements at the 10–14 level: influence of sample preparation: *Nucl. Instr. Meth. B* **266**, 4921–4926 (2008).
17 Braucher, R., et al., Preparation of ASTER in-house 10Be/9Be standard solutions: *Nucl. Instr. Meth. B* **361**, 335-340 (2015).
18 McMillan, P.F., Poe, B.T., Gillet, P., and Reynard, B. A study of $SiO_2$ glass and supercooled liquid to 1950 K via high-temperature Raman spectroscopy. *Geochim. Cosmochim. Acta* **58**, 3653-3664 (1994).
19 Bates, J.B. Raman spectra of α-cristobalite and β-cristobalite. *Journal of Chemical Physics* **57**, 4042-4047 (1972).
20 Masotta M., et al. 3D tomographic analysis reveals how coesite is preserved in Muong Nong-type tektites. *Nature Sci. Rep.* 10, 20608 https://doi.org/10.1038/s41598-020-76727-6 (2020).
21 Reagan, M.K., Morris, J.D., Eileen, H.A., Michael, M.T. Uranium series and beryllium isotope evidence for an extended history of subduction modification of the mantle below Nicaragua. *Geochim. Cosmochim. Acta* **58**, 4199-4212 (1994).
22 Rochette, p. et al. Magnetic properties and redox state of impact glasses: a review and new case studies from Siberia. *Geosciences*, *9*(**5**), 225, https://doi.org/10.3390/geosciences9050225 (2019)

Supplementary table 1: Sr and Nd isotopic results, including belizite data published in [7]

| sample | $^{147}Sm/^{144}Nd$ | 2σ | $^{143}Nd/^{144}Nd$ | 2σ | εNd | $^{87}Rb/^{86}Sr$ | 2σ | $^{87}Sr/^{86}Sr$ | 2σ | εSr |
|---|---|---|---|---|---|---|---|---|---|---|
| *P1* | *2.694* | *0.027* | *0.512772* | *0.000007* | *2.6* | *0.5931* | *0.0083* | *0.704736* | *0.000016* | *3.3* |
| *P6* | *2.687* | *0.027* | *0.512806* | *0.000006* | *3.3* | *0.8948* | *0.0125* | *0.705296* | *0.000025* | *11.3* |
| *P15* | *2.519* | *0.025* | *0.512960* | *0.000007* | *6.3* | *0.4640* | *0.0065* | *0.703714* | *0.000017* | *-11.2* |
| *P17* | *2.734* | *0.027* | *0.512917* | *0.000007* | *5.4* | *1.4048* | *0.0197* | *0.704913* | *0.000019* | *5.9* |
| *BZ-3* | | | *0.512850* | *0.000007* | *4.1* | | | *0.704574* | *0.000048* | *1.1* |
| *BZ-6* | | | *0.512858* | *0.000007* | *4.3* | | | *0.703952* | *0.000040* | *-7.8* |
| BZ[35] | | | | | *3.8* | | | *0.70402* | | *-6.8* |

Supplementary table 2: $^{10}$Be measurements with analytical error. Mean and standard deviation are reported for belizite, Pantasma soil and glass, with calculation of initial content at the $t_0$ time of impact (804 ka)

| *sample* | *10Be (Mat/g)* | *s.d. or error* |
|---|---|---|
| *BZ1* | *6.1* | *0.7* |
| *BZ2* | *4.4* | *0.4* |
| *BZ3* | *4.7* | *0.4* |
| *BZ4* | *6.3* | *0.7* |
| *BZ5* | *5.7* | *0.5* |
| *BZ6* | *8.2* | *1.1* |
| *mean* | *5.9* | *1.36* |
| *mean* $t_0$ | *9.08* | *2.09* |
| *P16* | *284.2* | *5.6* |
| *P17* | *295.2* | *5.6* |
| *mean* | *289.7* | *7.78* |
| *P1* | *0.37* | *0.06* |
| *P2* | *0.47* | *0.23* |
| *mean* | *0.42* | *0.07* |
| *mean* $t_0$ | *0.65* | *0.11* |

supplementary Fig.1: Belize glass finding site

a) field conditions for one belizite recovery in a ditch with Red Bank clay outcrop, along a modern grass field; b) closer view of the belizite in situ (size about 2 cm).

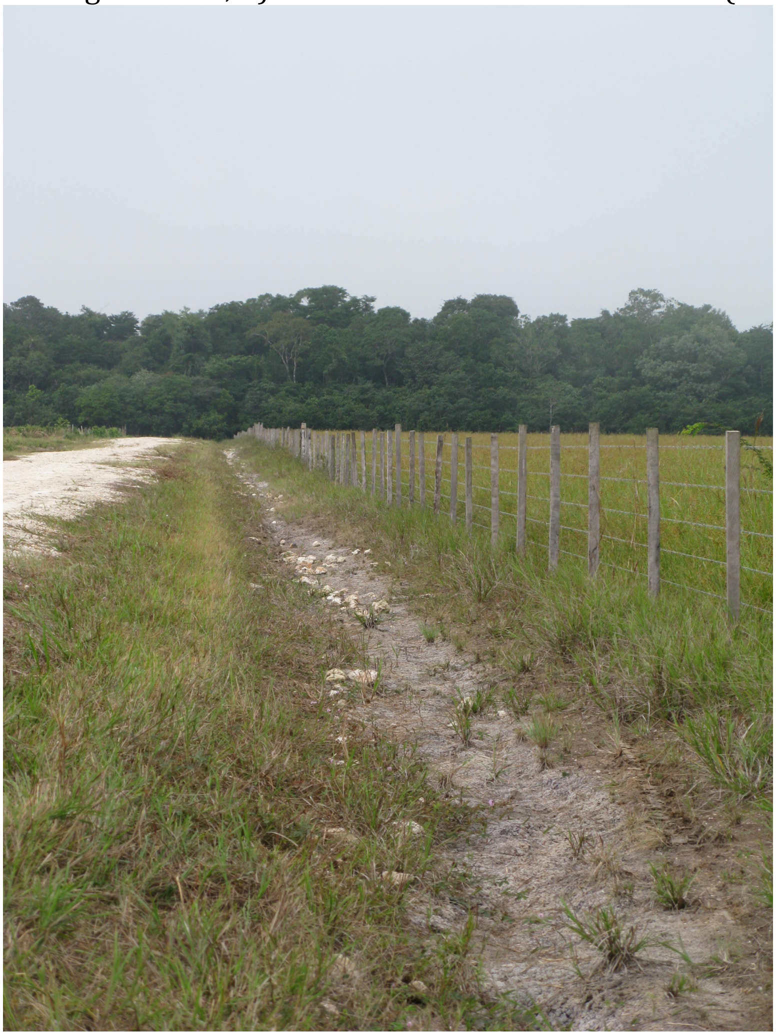

a)

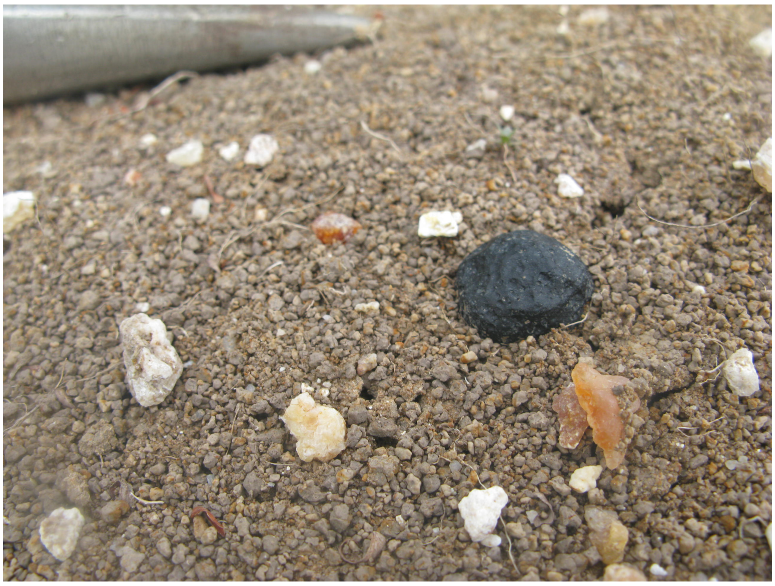

b)

supplementary Fig.2 Mass distribution of belizites compared to ivoirites

a) and b): mass distribution (in g) of belizite>10 g in JC collection, compared to ivoirites in our database[22]. Log-log fit allows to estimate the fractal dimension. To compare with usually published size distribution, one has to multiply this number by 3.

a)

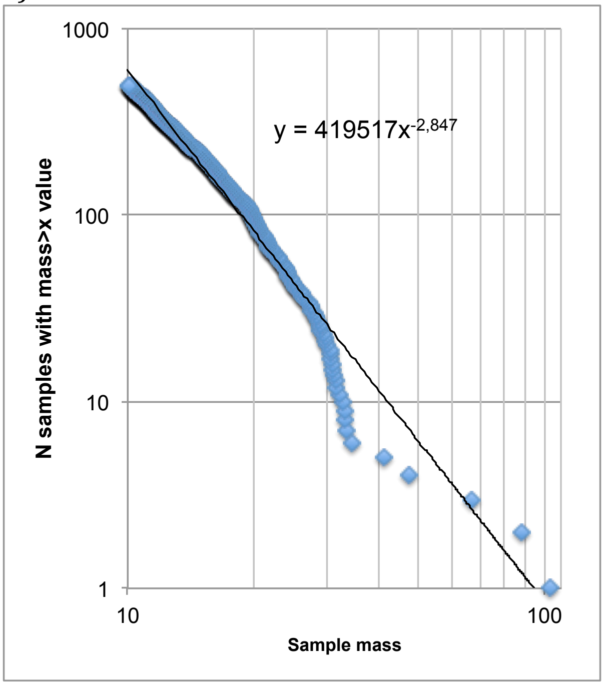


b)

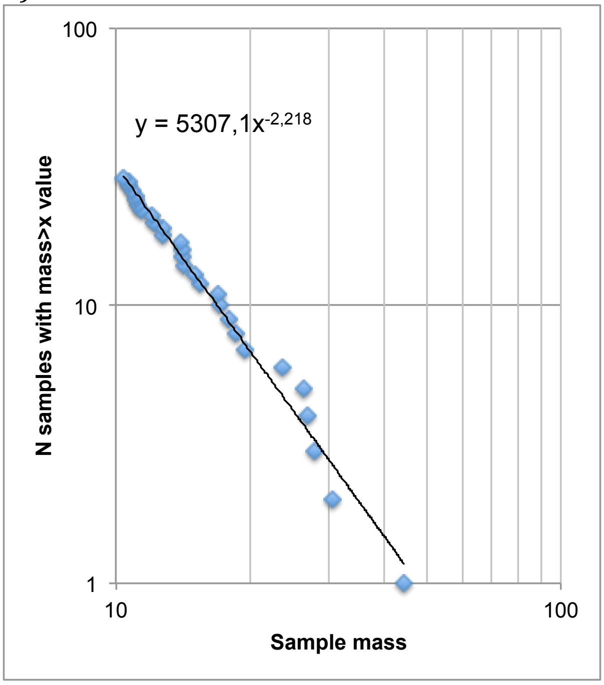

supplementary Fig.3: Tikal glass versus belizite composition

Element content in Tikal glass normalized to our average belizite (supplementary table 2). Elements are ordered by decreasing content

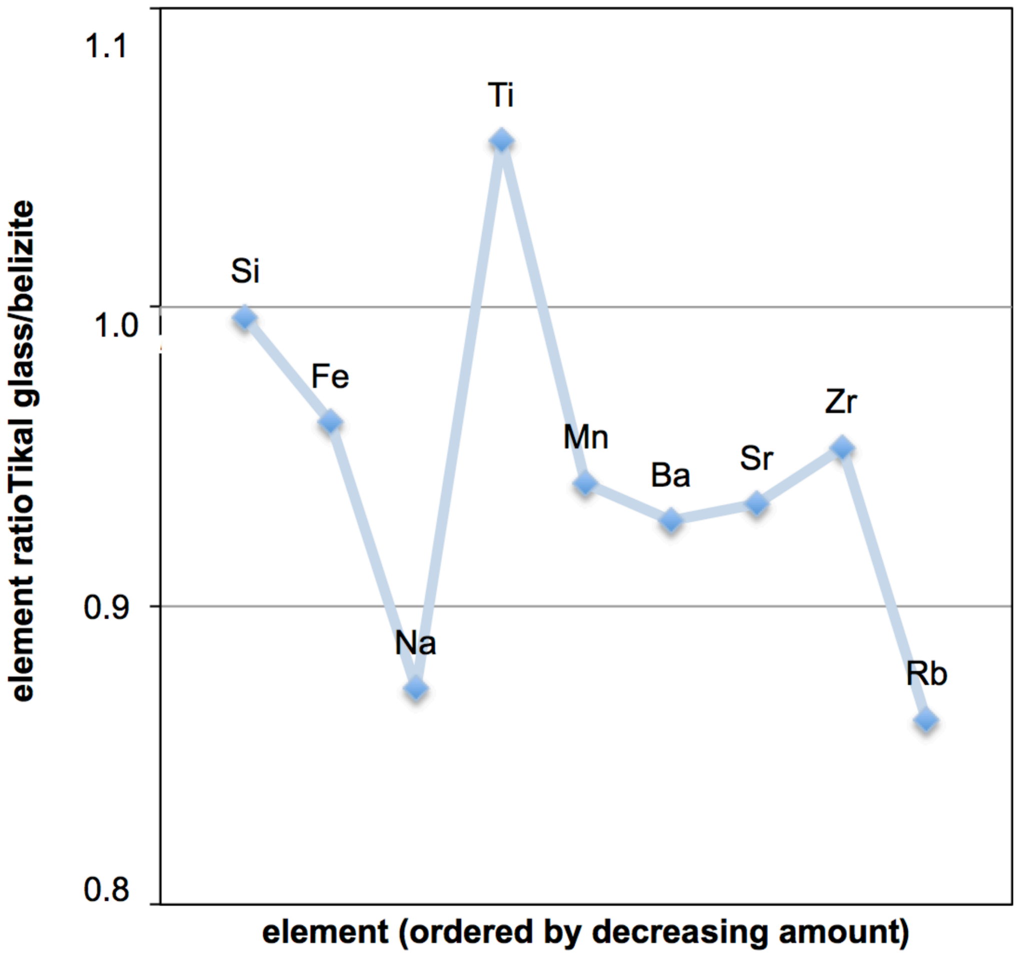

supplementary Fig.4: Raman spectroscopy results

Typical Raman spectra of the bulk tektite glass of complex composition, and of silica inclusions with typical Raman spectra of lechatelierite ($SiO_2$ glass) and $\alpha$-cristobalite.

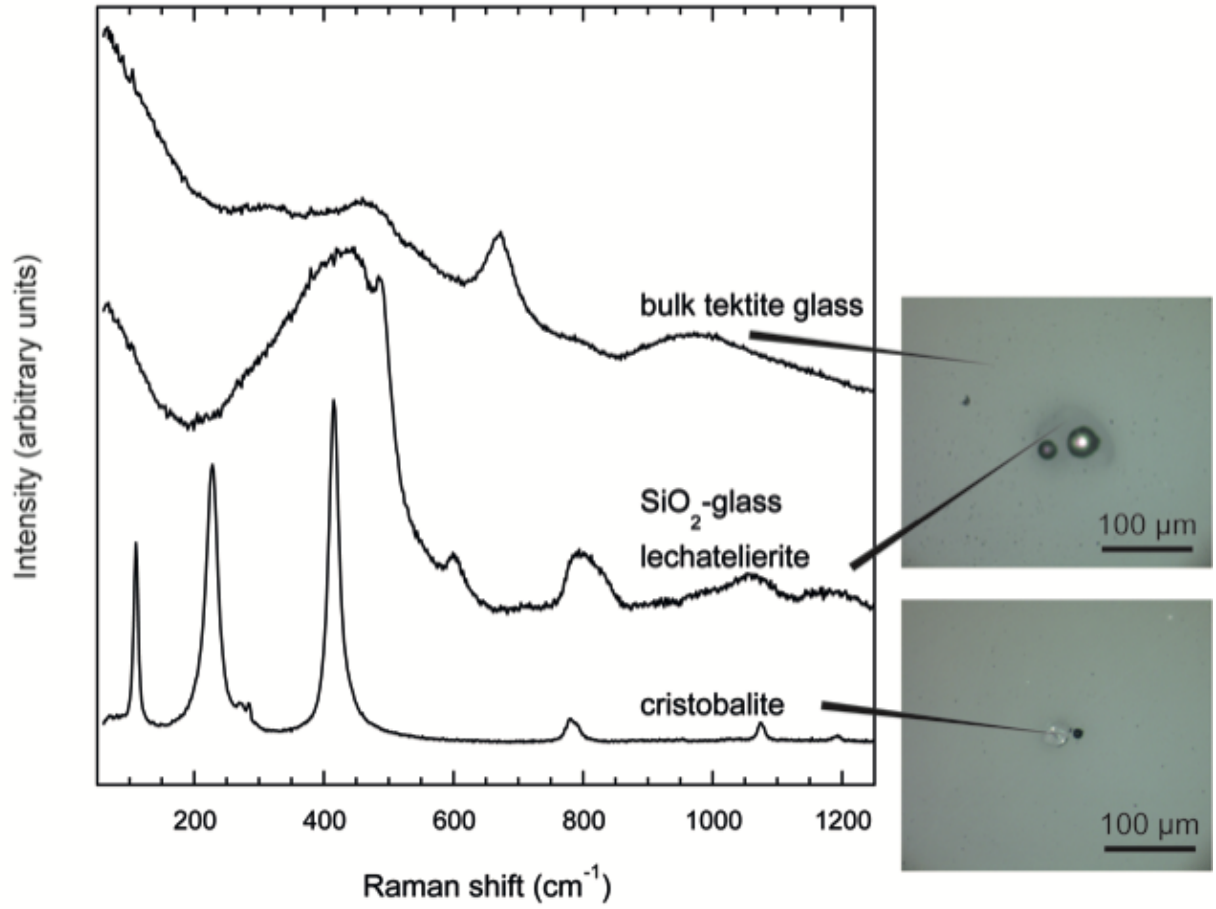

supplementary fig.5: selected Ar/Ar spectra and isochrones

selected Ar/Ar spectra and isochrones a) from Gif laboratory samples; b) from Curtin laboratory samples

a)

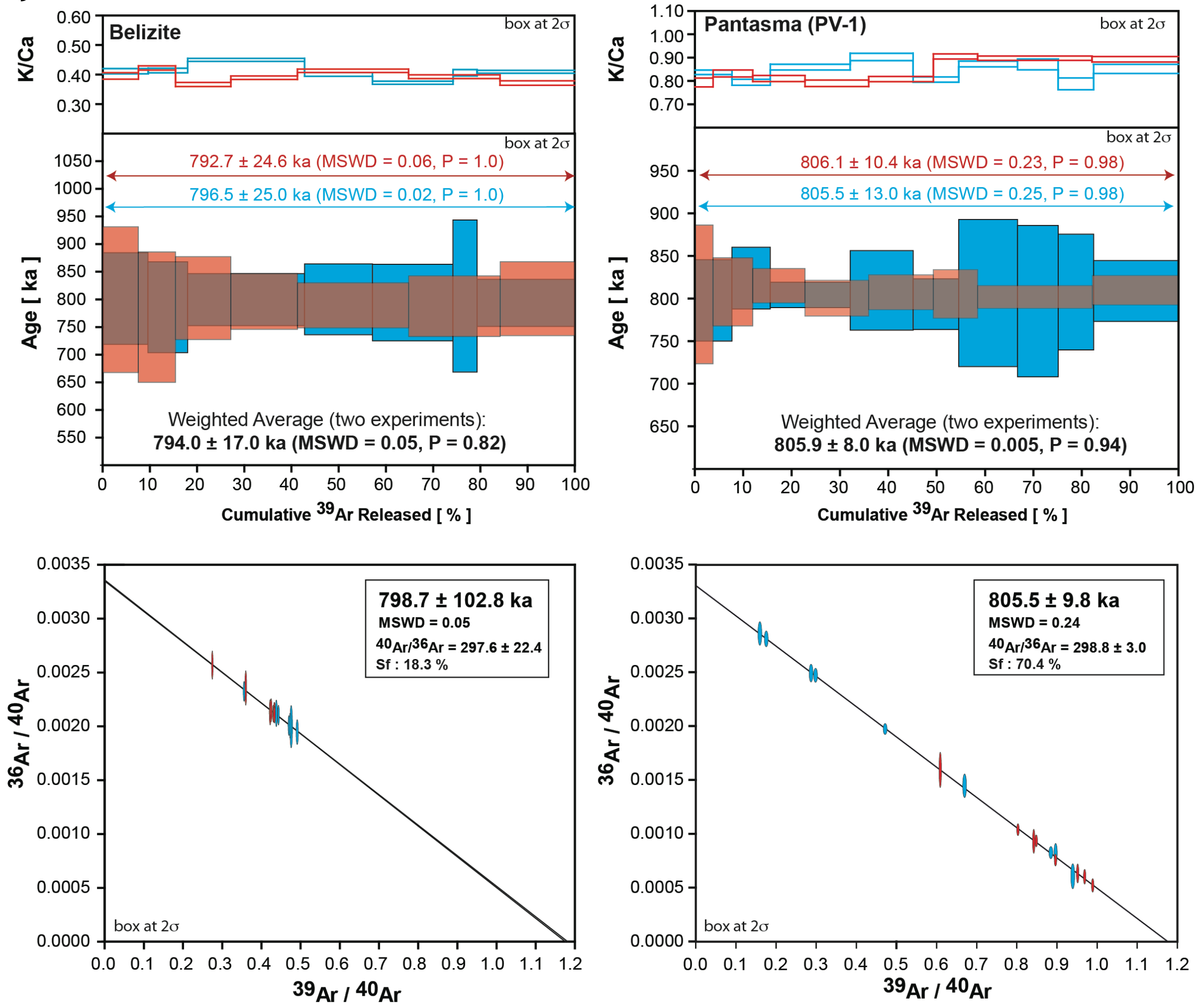

b)

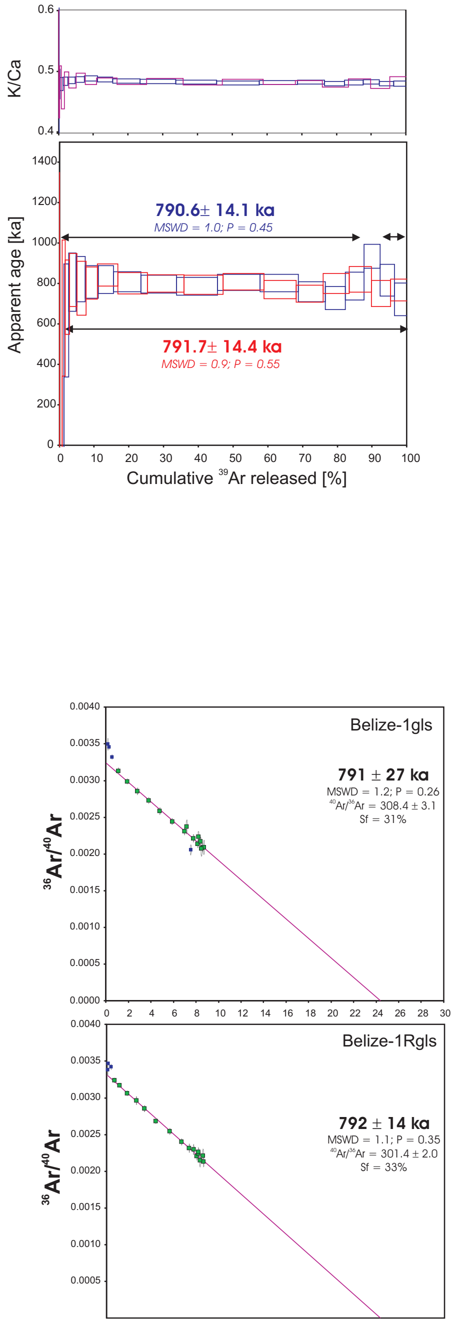
K/Ca
Apparent age [ka]
790.6± 14.1 ka
MSWD = 1.0; P = 0.45
791.7± 14.4 ka
MSWD = 0.9; P = 0.55
Cumulative $^{39}$Ar released [%]
Belize-1gls
791 ± 27 ka
MSWD = 1.2; P = 0.26
$^{40}$Ar/$^{36}$Ar = 308.4 ± 3.1
Sf = 31%
$^{36}$Ar/$^{40}$Ar
Belize-1Rgls
792 ± 14 ka
MSWD = 1.1; P = 0.35
$^{40}$Ar/$^{36}$Ar = 301.4 ± 2.0
Sf = 33%
$^{36}$Ar/$^{40}$Ar

supplementary fig.6: iron oxide inclusions in belizite and Pantasma glass

a) and b) FEG-SEM backscattered electron images of iron-oxyde inclusions in the strongly magnetic glass samples of belizite and Pantasma, respectively; b) after [22] Rochette, p. et al. Magnetic properties and redox state of impact glasses: a review and new case studies from Siberia. *Geosciences*, 9(5), 225, https://doi.org/10.3390/geosciences9050225 (2019).

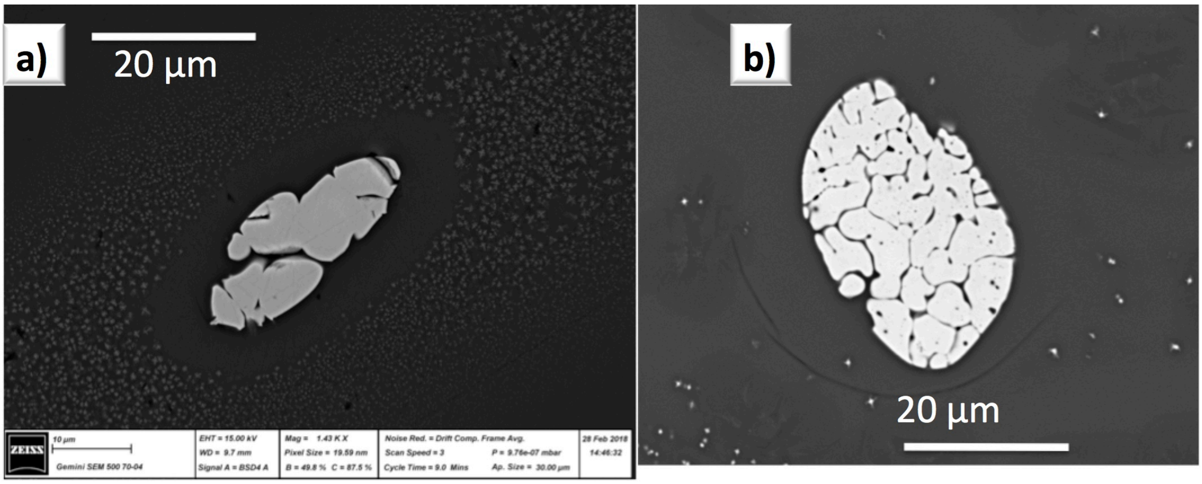

supplementary fig. 7: topography of Zhamanshin crater

SRTM topography for Zhamanshin crater, showing the published 14 km diameter circle. Real crater is likely the inner 6 km diameter circular depression.

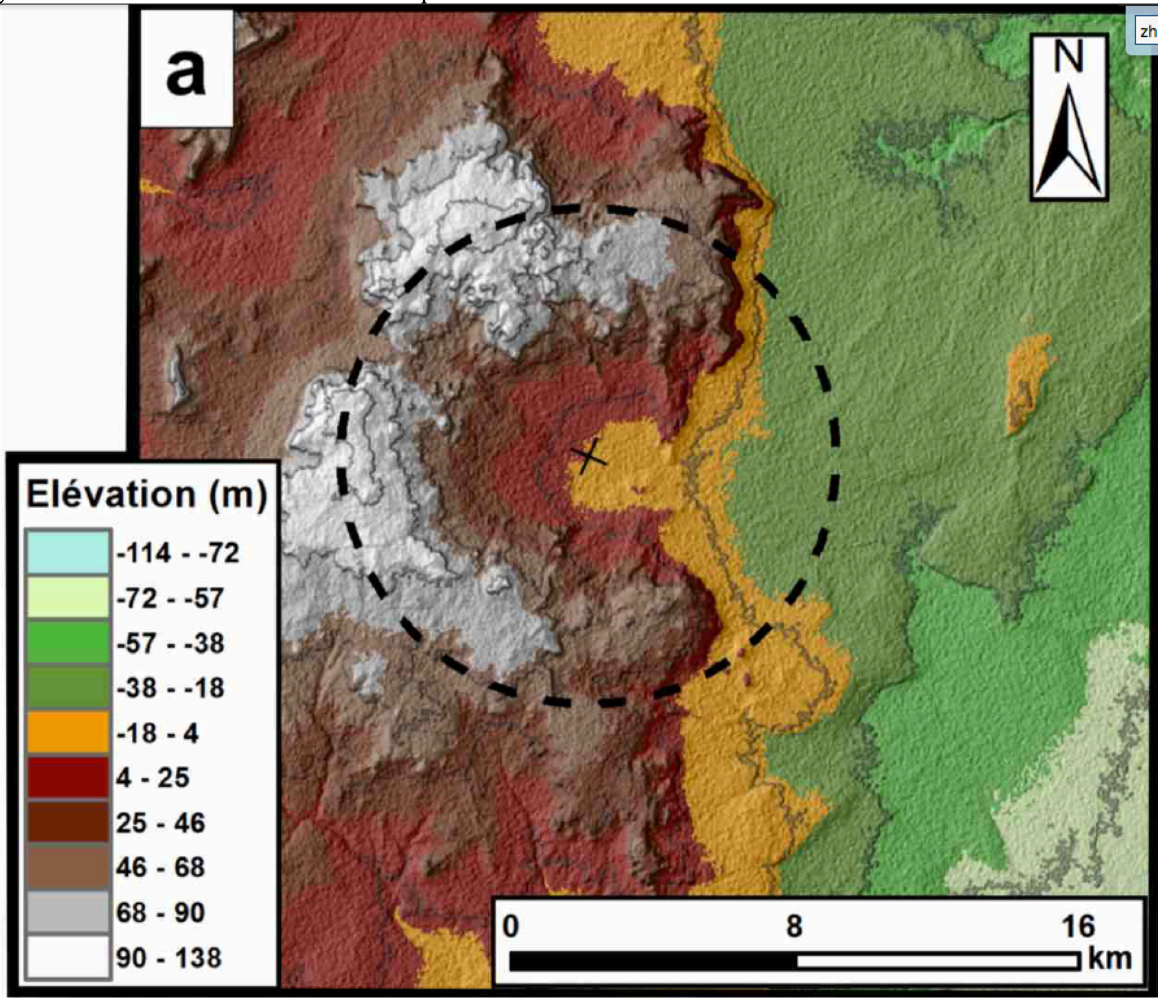

Supplement: Description of supplementary files [file EMS131201-supplement-Description_of_supplementary_files.pdf]
