# Supplementary material for: Impact glasses from Belize represent tektites from the Pleistocene Pantasma impact crater in Nicaragua
Source: Commun Earth Environ. Author manuscript; Available in PMC 2021 Aug 17. (PMC7611520; doi:10.1038/s43247-021-00155-1)
Supplement: Methods [file EMS131201-supplement-Methods.pdf]

Description of Additional Supplementary Files

File Name: Supplementary Data 1

Description: Ar/Ar detailed results

File Name: Supplementary Data 2

Description: Geochemical mean results in wt.% with relative standard deviation in % for major and ppm for minor elements (starting at Cr) of Pantasma impact glass (2 samples) and rocks (5 samples) compared to belizite (3 samples). For belizites is indicated either the absolute s.d. of individual measurements, or the detection limit (in bold) if s.d. was lower.
